# Amino acid intake and plasma concentrations and their interplay with gut microbiota in vegans and omnivores in Germany

**DOI:** 10.1007/s00394-021-02790-y

**Published:** 2022-01-16

**Authors:** Stefan Dietrich, Iris Trefflich, Per Magne Ueland, Juliane Menzel, Katharina J. Penczynski, Klaus Abraham, Cornelia Weikert

**Affiliations:** 1 Department of Food Safety, German Federal Institute for Risk Assessment (BfR), Berlin, Germany; 2 BEVITAL AS, Bergen, Norway; 3 Institute of Social Medicine, Epidemiology and Health Economics, Charité-Universitätsmedizin Berlin, Corporate Member of Freie Universität Berlin and Humboldt-Universität zu Berlin, Berlin, Germany

**Keywords:** Vegan diet, Vegans, Veganism, Dietary intake, Protein, Amino acids, Estimated average requirement, Microbiota

## Abstract

**Purpose:**

It has been estimated that most vegans meet the total protein requirements, but whether this is also true for individual essential amino acids (AAs) is unclear. Furthermore, a shift in protein intake is suggested to alter microbiota composition, but this association is unknown in terms of veganism or individual AAs. This cross-sectional study compared vegans and omnivores regarding dietary intake and plasma concentration of AAs. The prevalence of insufficient intake of essential AAs among vegans was determined using estimated average requirements (EAR) of WHO. Moreover, correlations between AAs intake and gut microbiota were investigated.

**Methods:**

Data of 36 vegans and 36 omnivores (30–60 years) were analysed. AA intake, AA plasma concentrations and gut microbiota were ascertained by three-day weighed food protocols, gas/liquid chromatography-tandem mass spectrometry and 16S rRNA sequencing, respectively.

**Results:**

At almost the same energy intake, the intake of 9 AAs in vegans was significantly lower than in omnivores, with median differences of − 27.0% to − 51.9%. However, only one female vegan showed total protein and lysine intake below the EAR. Vegans showed lower lysine (− 25.0%), but higher glycine (+ 25.4%) and glutamate (+ 13.1%) plasma concentrations than omnivores. Correlation patterns between AA intake and bacterial microbiota differed between vegans and omnivores. In vegans 19 species and in omnivores 5 species showed correlations with AA intake.

**Conclusion:**

Vegans consumed apparently sufficient but lower AAs than omnivores. In addition, the different AAs intake seems to influence the microbiota composition. The use of short-term dietary data without considering usual intake limits these findings.

**Supplementary Information:**

The online version contains supplementary material available at 10.1007/s00394-021-02790-y.

## Introduction

A vegan diet, which excludes the consumption of all animal products, is becoming increasingly popular in Western countries for ethical, environmental and health-related reasons [1]. Apart from these positive aspects, a vegan diet may also poses the risk of nutritional deficiencies [2, 3]. For instance, it is widely believed that a vegan diet is associated with an insufficient dietary intake of total protein and consequently also with an insufficient intake of essential amino acids (AAs). Essential AAs are required for many important functions in the human body, e.g. for the synthesis of metabolites that are involved in signalling, cell growth, gene expression and repair processes [4]. Since a long-term undersupply of essential AAs is associated with a negative health status, the World Health Organization (WHO) has published Estimated Average Requirements (EAR) as a guidance for the daily intake of total protein and essential AAs [5]. Some studies have reported that most vegans have total protein intakes which are in line with the EAR [6], but whether this is also the case for individual essential AA is largely unknown. Findings from the European Prospective Investigation into Cancer and Nutrition (EPIC) Oxford study suggest that vegans have an up to 47% lower intake of essential AAs than omnivores, which was also partly reflected in the AA plasma concentrations [7]. However, in this or other study populations, it has not yet been investigated whether the dietary intake of essential AAs among vegans is sufficient and complies with the EAR of WHO [6–9].

In this context, the question arises whether a potential lower intake of AAs in vegans compared to omnivores affects the composition of intestinal microbiota. Recent scientific evidence suggests that long-term altered dietary protein intake modulates gut microbiota composition and diversity which may subsequently impact human health [10–13]. Moreover, gut microbiota and their metabolic products also seem to depend on the ratio of consumed food of plant and animal origin [14]. Diets high in carbohydrate and fibre have been associated with higher abundance of *Lachnospiraceae*, *Ruminococcaceae* and *Bifidobacteria*, and a lower abundance of *Bacteroides* [14]. However, the bacterial fermentation of carbohydrate and fibre occurs mainly in the proximal colon, so that the amount of available fibre decreases towards the distal colon, where proteolytic fermentation becomes more important [14]. Indeed, of the consumed dietary proteins, significant amounts escape digestion and absorption in the upper digestive tract and reach the distal colon [15]. Proteolytic fermenter, like *Bacteroides*, *Alistipes* and *Parabacteroides,* are known to utilize AAs not only for their metabolism but also for synthesis of secondary metabolites, including ammonium and short chain fatty acids [14]. In particular, the abundance of *Bacteroides* has been found to increase with higher meat consumption [14]. However, to the best of our knowledge, it has not yet been investigated whether potential differences in the dietary intake of individual AAs, from a vegan diet compared to an omnivorous diet, modulate gut microbiota, particularly with regard to the abundance of protective or harmful taxa.

The present study aimed to compare the dietary intake and blood plasma concentrations of AAs in participants following a vegan or omnivorous diet. Furthermore, the prevalence of potentially inadequate intake of essential AAs among vegans was determined using EAR of WHO [5] as cut-off point. It was also examined whether correlation patterns between the dietary intake of AAs and the abundance of gut bacterial microbiota differ between the two dietary groups using Spearman partial correlation adjusted, among others, for dietary intake of fat, carbohydrate and fibre.

## Methods

### Study population

The cross-sectional study “Risks and Benefits of a Vegan Diet” (RBVD) included 36 vegans and 36 omnivores (50% women and men each), aged 30–60 years, who lived in Berlin (Germany). In a phone screening, it was checked whether the participants had been followed their diet for at least one year and whether their diet corresponded to the RBVD study definition of vegan or omnivorous diet [16]. A vegan diet was defined as non-consumption of any animal food products. An omnivorous diet (regular meat-eaters) should contain at least three portions of meat per week or two portions of meat and two portions of processed meat (e.g. cold cuts, sausages) per week. Exclusion criteria were BMI ≥ 30 kg/m^2^, pregnancy, breastfeeding, current infection and serious prevalent illness, including cardiovascular disease and cancer. Participants in the two dietary groups were matched by sex and age and visited the study centre twice. During the first visit, the participants gave their written informed consent, received instructions to document their diet using a three-day weighed food protocol; at the second visit, their anthropometry, lifestyle and medical conditions were assessed and a blood sample was taken [17]. This study was conducted at the German Federal Institute for Risk Assessment (Berlin, Germany), in accordance with the Declaration of Helsinki, and was approved by the Ethics Committee of Charité University Medical Center Berlin (No. EA4/121/16).

The RBVD study was designed to investigate the association of a vegan diet on bone health compared to an omnivorous diet. Power calculation (significance of 5% and a power of 80%) resulted in a total of 72 participants (36 vegans, 36 omnivores) [18].

### Assessment of dietary amino acid intake

The dietary intake was recorded in a three-day weighed food protocol, which covered two weekdays and one day at the weekend within one to four weeks before the second visit. The amount of AAs and macronutrients in the consumed food from the three-day weighed food protocol was determined using the German Nutrient Database [19]. The German Nutrient Database is a national food composition database containing the nutrient values of almost 15,000 commercially available foods. On this basis, the dietary intake of 18 AAs in mg per kg of body weight per day was calculated. However, for some new vegan food items (e.g. vegan spread or vegan cakes) only the nutrient amount of the total protein was available from the database, but not for the nutrient amount of the individual AAs. The protein percentage of these items on the recorded total protein amounts to approximately 4% in vegans. Please also note, when analysing the nutrient composition of foods in the laboratory, as used for the German Nutrient Database, asparagine and glutamine are transformed into aspartate and glutamate, respectively. Hence, for asparagine and aspartate as well for glutamine and glutamate only the combined dietary intake could be determined and presented here.

### Measurements of plasma concentrations of amino acid

At the study centre, 60 mL of venous blood was collected from the participants on the morning of the second visit. All participants fasted overnight. Blood samples were fractionated and aliquoted into serum, EDTA plasma and erythrocytes, and stored in freezers (− 80 °C) until analysis. Blood plasma samples were used to analyse concentrations of AA. The analysis was conducted at BEVITAL Laboratory in Bergen, Norway. Plasma concentrations of arginine were measured by liquid chromatography-tandem mass spectrometry [20]. Concentrations of the remaining AAs were measured by gas chromatography-tandem mass spectrometry [21, 22]. Limits of detection and coefficients of variability were reported elsewhere [20–22].

### Gut microbiota analysis

Faecal sample collection and microbiota analyses were described in detail before [23]. In short, the participants collected an entire faecal sample at home on the morning of the second visit to the study centre or on the day after. At study centre, faecal samples were homogenized, aliquoted and frozen at − 80° until further analysis. The time between defecation and processing at the study site was less than four hours [24]. Microbiota profiling was performed by 16S ribosomal RNA (rRNA) gene sequencing by CeMeT GmbH (Tübingen, Germany). After sequencing, 16S rRNA sequence data were matched with the NCBI Bacterial 16S rRNA database. The taxonomic classification was conducted with MALT [25], resulting in the identification of Operational Taxonomic Units.

Microbiota data were available for all 72 participants. Overall, 27 phyla, 48 classes, 226 families, 687 genera and 1195 species were identified in the participants’ faecal samples [26]. For statistical analysis, only the most abundant representatives of each taxon were included by using the following criteria: (1) present in at least 50% of participants on a vegan or an omnivorous diet and (2) with an absolute abundance of 100 reads in at least one participant on the given taxonomic level. The final data set for the analyses thus consisted of 8 phyla, 16 classes, 34 families, 46 genera and 50 species. The BioVision Ammonia Colorimetric Assay Kit II was used to measure ammonium levels from faecal samples at the German Institute of Human Nutrition Potsdam-Rehbruecke (Germany).

### Statistical analysis

Participants’ characteristics, nutrient intakes and biomarkers were reported for the two dietary groups using mean and standard deviation (SD) for normally distributed variables, median and interquartile range (IQR) for skewed variables, and relative percentages for categorical variables. Differences between vegans and omnivores were tested using Kruskal–Wallis test for continuous variables. The dietary intake and plasma concentrations of AAs were mostly non-normally distributed. Resulting p values for differences in dietary AA intake and plasma AA concentrations were corrected for multiple testing using Bonferroni correction. The median difference (*x*) was calculated as follows: *x* = (median 1 – median 2)/median 2*100. The prevalence of inadequate intake was calculated as the proportion of individuals whose intake was less than the EAR of WHO. To estimate the prevalence of inadequate intake we excluded potential under-reporter. Potential under-reporters were identified according to published cut-offs [27]. The cut-offs were computed based on the individual relation of the recorded total energy intake to the estimated basal metabolic rate [28] and assuming a moderate physical activity level [29].

Spearman partial correlation analyses, adjusted for potential confounders, were used to investigate correlation patterns of the dietary intake of AAs with plasma concentrations of AAs and with gut microbiota. The following potential confounders were considered after literature screening for correlation analyses: age (years), sex (men/women), body mass index (BMI; kg/m^2^), physical activity (PA; h/week), smoking status (never, former, current), alcohol intake (g/d), faecal pH, AA supplementation (yes/no), antibiotic medication during the previous two months (yes/no), under-reporter (yes/no) and intake of fibre (g/day), carbohydrates (g/day) and fat (g/day). Heatmaps were used to visualize significantly tested partial correlation coefficients (*p* < 0.05). However, due to the small sample size and the high number of tests, none of the partial correlation coefficients was significant after correction for multiple testing. All analyses were performed using SAS (version 9.4) and R (version 3.6.3).

## Results

The distribution of general characteristics (Table 1) was almost the same between vegans and omnivores. No significant differences in smoking status or alcohol consumption (*p* > 0.05) were observed; however, the proportion of current smokers and consumption of alcohol was slightly higher in omnivores than in vegans. The median duration of veganism was 4.8 years. One vegan and two omnivores used AA supplements. The faecal pH and ammonium concentrations were lower in vegans than in omnivores. Vegans had a higher intake of fibre and carbohydrates as well as a lower intake of protein and fat than omnivores (Table 2), at almost the same energy intake. No linear trend was observed for muscle and fat mass, PA, ammonium concentration and faecal pH across tertiles of protein intake in vegans or omnivores (Supplemental Table S1).

**Table 1:** Characteristics of the study population in the RBVD study

Characteristics	Vegans^a^ (*n* = 36)	Omnivores^a^ (*n* = 36)
Women, *n* (%)	18 (50)	18 (50)
Age (years)	37.5 (32.5–44.0)	38.5 (32.0–46.0)
BMI (kg/m^2^) ^a^	22.9 ± 3.2	24.0 ± 2.1
Physical activity (h/week)	2.8 (0.9–3.8)	2.3 (1.2–4.1)
Duration vegan diet (years)	4.8 (3.1–8.7)	–
Alcohol consumption (g/d)	0.1 (0–3.3)	1.3 (0–11.2)
Education, *n* (%)		
Low	0 (0.0)	1 (2.8)
Intermediate	11 (30.6)	11 (30.6)
High	25 (69.4)	24 (66.7)
Smoking status, *n* (%)		
Non-smoker	24 (66.7)	21 (58.3)
Ex-smoker	8 (22.2)	6 (16.7)
Smoker	4 (11.1)	9 (25.0)
Energy intake (kcal/d)	2270 (1800–2762)	2386 (2081–2737)
Supplementation overall, *n* (%)	35 (97.2)	12 (33.3)
AA supplementation, *n* (%)	1 (2.8)	3 (8.3)
Faecal ammonium concentration (µg/g)	501.4 ± 228.5	647.7 ± 333.6
Faecal pH-value	6.41 ± 0.48	6.73 ± 0.45

^a^Data are reported as percentage, mean ± SD for normally distributed or median (IQR) for skewed variables

*AA* amino acid, *BMI* body mass index

**Table 2:** Dietary data of participants with vegan and omnivorous diet in the RBVD study

Characteristics	Data with under-reporters		Data without under-reporters	
	Vegans^a^ (*n* = 36)	Omnivores^a^ (*n* = 36)	Vegans^a^ (*n* = 32)	Omnivores^a^ (*n* = 34)
Energy intake (kcal/d)	2270 (1800–2762)	2386 (2081–2737)	2335 (1939–2870)	2395 (2159–2750)
Dietary intake				
Protein, % of energy	13.5 ± 3.7	15.4 ± 4.0	13.8 ± 3.8	14.9 ± 3.0
Fat, % of energy	34.2 ± 8.2	41.4 ± 7.5	35.0 ± 8.0	41.6 ± 7.2
Carbohydrates, % of energy	49.8 ± 8.2	40.4 ± 8.1	49.2 ± 8.1	40.8 ± 7.4
Protein (g/kg body weight per day)	1.0 (0.9–1.4)	1.2 (1.0–1.5)	1.1 (0.9–1.5)	1.2 (1.1–1.4)
Fat (g/kg body weight per day)	1.2 (0.9–1.5)	1.5 (1.2–1.8)	1.2 (1.1–1.6)	1.5 (1.3–1.9)
Carbohydrates (g/kg body weight per day)	4.2 (3.2–4.9)	3.2 (2.9–3.7)	4.2 (3.6–5)	3.3 (3–3.7)
Fibre(g/kg body weight per day)	0.7 (0.5–0.9)	0.3 (0.3–0.4)	0.7 (0.5–0.9)	0.3 (0.3–0.4)

^a^Data are reported as percentage, mean ± SD for normally distributed or median (IQR) for skewed variables

### Dietary intake of amino acids

The dietary intake (Table 3) of nine of 18 AAs differed significantly between vegans and omnivores after correction for multiple testing. Compared to omnivores, vegans had a lower median intake of the essential AAs isoleucine by 33.4%, leucine by 34.4%, lysine by 48.0%, methionine by 51.9%, threonine by 32.6% and valine by 35.5%; of the semi-essential AA histidine by 37.5%; and of the non-essential AAs proline by 27.0% and tyrosine by 37.2%. A trend for lower median intake by 26.6% was observed for tryptophan.

**Table 3:** Dietary intake of amino acids in the RBVD study

Amino acids	Vegans^a^ (*n* = 36)	Omnivores^a^ (*n* = 36)	Median difference [%]	Raw *p* value^b^	Corrected *p* value^b,c^
Essential and semi-essential amino acids [mg/d per kg body weight]					
Histidine	20.0 (17.4–31.1)	32.0 (27.5–39.0)	− 37.5	< 0.001	** < 0.001**
Isoleucine	37.3 (31.7–56.0)	56.0 (47.6–68.3)	− 33.4	< 0.001	**0.002**
Leucine	62.6 (51.8–91.8)	95.5 (79.2–113.5)	− 34.4	< 0.001	**0.001**
Lysine	41.0 (32.2–67.1)	78.9 (63.0–97.5)	− 48.0	< 0.001	** < 0.001**
Methionine	12.5 (9.5–17.0)	26.0 (22.1–31.4)	− 51.9	< 0.001	** < 0.001**
Phenylalanine	42.2 (33.4–62.0)	54.7 (45.3–64.2)	− 22.9	0.015	0.30
Threonine	31.9 (25.7–48.3)	47.3 (39.5–58.5)	− 32.6	< 0.001	**0.003**
Tryptophan	10.5 (9.0–14.8)	14.3 (12.1–16.5)	− 26.6	0.004	0.091
Valine	43.7 (37.6–65.7)	67.7 (56.7–76.8)	− 35.5	< 0.001	**0.002**
Total	300.9 (251.2–454.1)	476.9 (395.1–564.5)	− 36.9	< 0.001	**0.001**
Non-essential amino acids [mg/d per kg body weight]					
Alanine	57.2 (40.8–71.4)	60.7 (49.1–79.7)	− 5.8	0.13	1
Arginine	60.7 (45.3–87.2)	65.5 (51.7–76.8)	− 7.3	0.69	1
Aspartate/Asparagine	86.8 (68.4–134.7)	103.7 (83.7–124.4)	− 16.3	0.11	1
Cysteine	15.9 (12.2–20.3)	16.1 (13.8–19.3)	− 1.2	0.71	1
Glutamate/Glutamine	206.7 (161.4–269.5)	250.1 (225.3–286.4)	− 17.6	0.02	0.46
Glycine	41.1 (32.2–57.0)	45.6 (40.3–61.3)	− 9.9	0.05	1
Proline	65.4 (51.2–86.7)	89.6 (77.1–102.7)	− 27.0	< 0.001	**0.001**
Serine	45.6 (36.7–65.8)	61.4 (52.0–68.5)	− 25.7	0.007	0.13
Tyrosine	28.0 (22.3–40.3)	44.6 (35.3–51.4)	− 37.2	< 0.001	** < 0.001**
Total	616.7 (475.6–807.2)	732.3 (650.2–871.0)	− 15.8	0.02	0.38

^a^Data are reported as median (IQR)

^b^Differences between groups were tested with Kruskal–Wallis test

^c^Bonferroni correction was applied to correct *p* values for multiple testing (raw *p* value multiplied by 20), bold *p*-values indicates statistical significance

To consider potential under-reporting, a sensitivity analysis was conducted with exclusion of four vegans and two omnivores. In the sensitivity analysis, nearly the same differences between the two diet groups in terms of medians of AAs were observed (Supplemental Table S2).

### Prevalence of inadequate intake according to EAR of WHO

Despite the lower intake of nine AAs among vegans, the data without under-reporters indicate that the median dietary intake of total protein and all essential AAs in vegans was above the EAR of WHO (Table 4). Only one female vegan showed a dietary intake of total protein (0.62 g/kg body weight per day) and of lysine (23.0 mg/kg body weight per day) that was below the EAR of WHO (Table 4). This led to a prevalence of inadequate intake of total protein and lysine of 2.8%. For the other essential AAs the prevalence of inadequate intake was zero for the data without under-reporters (Table 4). Among the four excluded under-reporters with a vegan diet, three had an individual intake of total protein, lysine, leucine and valine, two of total sulphur, and one of isoleucine and threonine, which was below the EAR (Supplemental Table S3). No participant with an omnivorous diet showed an intake of total protein or essential AAs, which was below the EAR (data not shown).

**Table 4:** Prevalence of inadequate intakes of amino acids among vegans without under-reporters (*n* = 32) in the RBVD study in relation to the estimated average requirements (EAR) of WHO

Amino acids	EAR [mg/kg body weight per day]^a^	Dietary intake [mg/kg body weight per day]^b^	Prevalence of inadequate intakes excluding under-reporters [% (*n*)]
Histidine	10.0	20.6 (18.9–32.7)	0 (0)
Isoleucine	20.0	40.0 (34.0–60.5)	0 (0)
Leucine	39.0	65.3 (56.0–97.9)	0 (0)
Lysine	30.0	41.8 (34.2–69.1)	2.8 (1)
SAA	15	28.9 (25.8–38.6)	0 (0)
AAA	25.0	72.1 (62.1–110.0)	0 (0)
Threonine	15.0	33.2 (28.5–49.9)	0 (0)
Tryptophan	4.0	11.3 (9.8–15.5)	0 (0)
Valine	26.0	45.5 (40.2–69.7)	0 (0)
Total Protein [g/kg per day]	0.66	1.03 (0.89–1.36)	2.8 (1)

^a^As reported in chapter 8, Table 23 [5]

^b^Data are reported as median (IQR)

*AAA* aromatic amino acids (phenylalanine and tyrosine), *EAR* estimated average requirements, *SAA* sulphur amino acids

### Plasma concentrations of amino acids

The observed differences in dietary intake of AAs were hardly reflected in plasma concentrations of AAs (Table 5). In vegans, the median plasma concentration of lysine was 25% lower than in omnivores. For tryptophan, a trend for 12.7% lower median concentrations among vegans was observed as well. In contrast, vegans had a 13.1% and 25.4% higher median plasma concentration of glutamine and glycine than omnivores. For serine, a trend for 14.2% higher median concentrations among vegans was observed as well. Significant differences for other AAs were not observed.

**Table 5:** Plasma amino acid concentrations in the RBVD study

Amino acids	Vegans^a^ (*n* = 36)	Omnivores^a^ (*n* = 36)	Median difference [%]	Raw *p* value^b^	Corrected *p* value^b,c^
Essential and semi-essential amino acid concentrations [µmol/L]					
Histidine	86.7 (79.4–93.2)	81.9 (73.4–89.0)	+ 5.5	0.04	0.74
Isoleucine	67.0 (54.9–72.6)	63.1 (56.0–72.7)	+ 5.8	0.95	1
Leucine	117.5 (103.6–137.0)	120.0 (114.4–143.7)	− 2.1	0.07	1
Lysine	128.5 (119.0–147.7)	171.4 (152.3–189.3)	− 25.0	< 0.0001	** < 0.0001**
Methionine	26.7 (24.3–30.4)	26.8 (25.9–29.9)	− 0.4	0.32	1
Phenylalanine	58.3 (52.1–63.2)	59.3 (55.7–63.5)	− 1.7	0.42	1
Threonine	126.0 (108.7–141.3)	129.6 (117.9–160.0)	− 2.8	0.33	1
Tryptophan	65.5 (59.1–74.7)	75.0 (66.9–82.2)	− 12.7	0.004	0.08
Valine	223.2 (208.7–258.0)	246.8 (220.1–281.5)	− 9.6	0.02	0.40
Non-essential amino acid concentrations [µmol/L]					
Alanine	371.4 (292.1–448.2)	342.3 (311.2–382.2)	+ 7.8	0.29	1
Arginine	64.1 (52.7–74.4)	69.1 (59.0–76.0)	− 7.2	0.35	1
Asparagine	64.4 (58.8–78.3)	60.0 (55.3–63.9)	+ 6.8	0.01	0.13
Aspartic acid	2.8 (2.4–3.5)	3.0 (2.4–3.6)	− 6.7	0.64	1
Cysteine	280.9 (263.9–301.8)	274.3 (250.5–299.5)	+ 2.3	0.37	1
Glutamine	635.6 (568.1–694.9)	552.5 (513.4–582.3)	+ 13.1	< 0.0001	**0.001**
Glutamic acid	31.2 (19.6–42.0)	35.2 (22.9–45.9)	− 11.4	0.29	1
Glycine	329.2 (279.3–385.0)	245.5 (230.4–303.0)	+ 25.4	< 0.0001	**0.001**
Proline	174.7 (146.5–244.4)	174.6 (139.1–195.7)	+ 0.1	0.24	1
Serine	132.6 (117.2–150.9)	113.8 (102.0–127.9)	+ 14.2	0.003	0.06
Tyrosine	55.1 (48.2–62.6)	57.2 (52.7–69.3)	− 3.7	0.18	1

^a^Data are reported as median (IQR)

^b^Differences between groups were tested with Kruskal–Wallis test

^c^Bonferroni correction was applied to correct *p* values for multiple testing, bold *p*-values indicates statistical significance

### Correlations between the dietary intake and plasma concentrations of amino acids

**Fig. 1:**
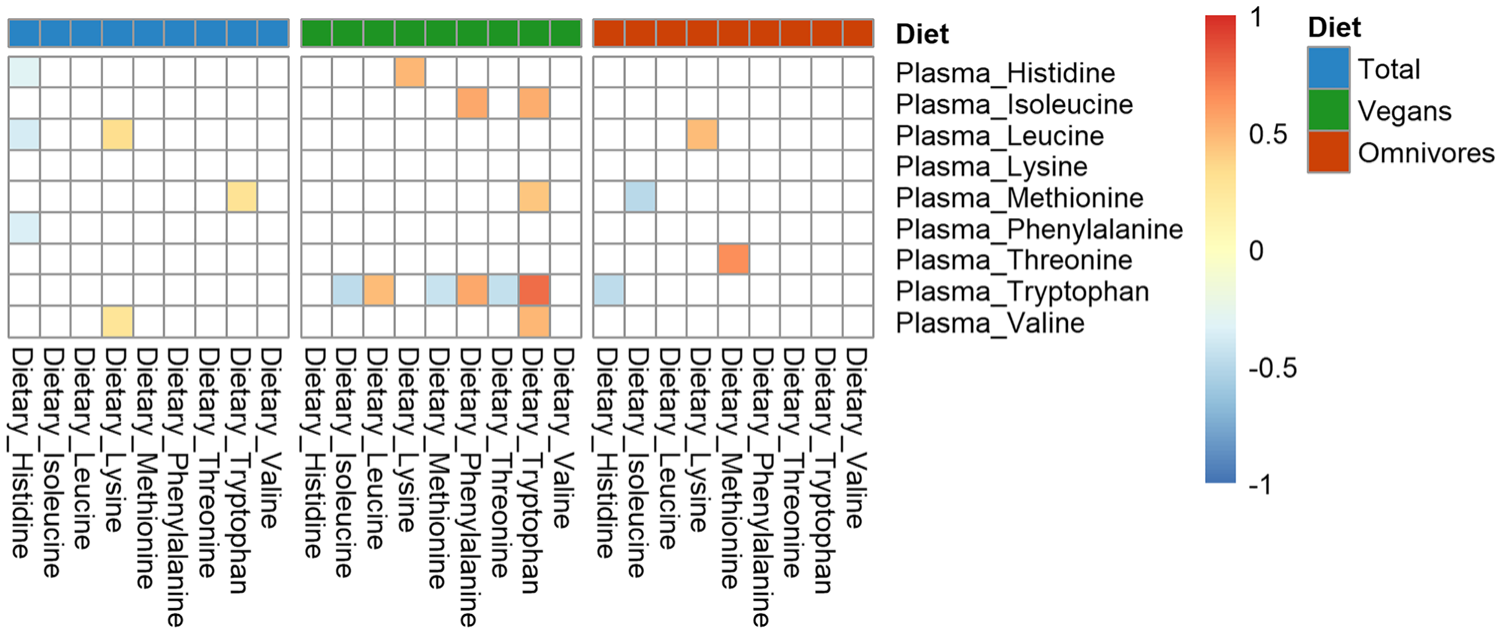
Partial Spearman correlation between dietary intake and plasma concentrations of essential amino acids. Partial Correlations were adjusted for age, sex, body mass index, physical activity, smoking status, alcohol consumption, amino acid supplementation and under-reporting. Data of the 36 vegans and 36 omnivores were analysed. The colours in the figure represent partial correlation coefficients from − 1 to 1. Shown are only correlation coefficients with uncorrected *p* values < 0.05

For most of the essential AAs, the dietary intake was not correlated with their respective plasma concentration in vegans and omnivores (Fig. 1). Only the dietary intake of tryptophan was positively correlated (*r* = 0.77, *p* =  < 0.001) with the plasma concentrations of tryptophan in vegans. In vegans, the plasma concentration of tryptophan was also significantly correlated with the dietary intake of isoleucine (*r* = − 0.46), leucine (*r* = 0.47), methionine (*r* = − 0.43), phenylalanine (*r* = 0.55) and threonine (*r* = − 0.46).

### Dietary intake of AA and abundance of microbiota

**Fig. 2:**
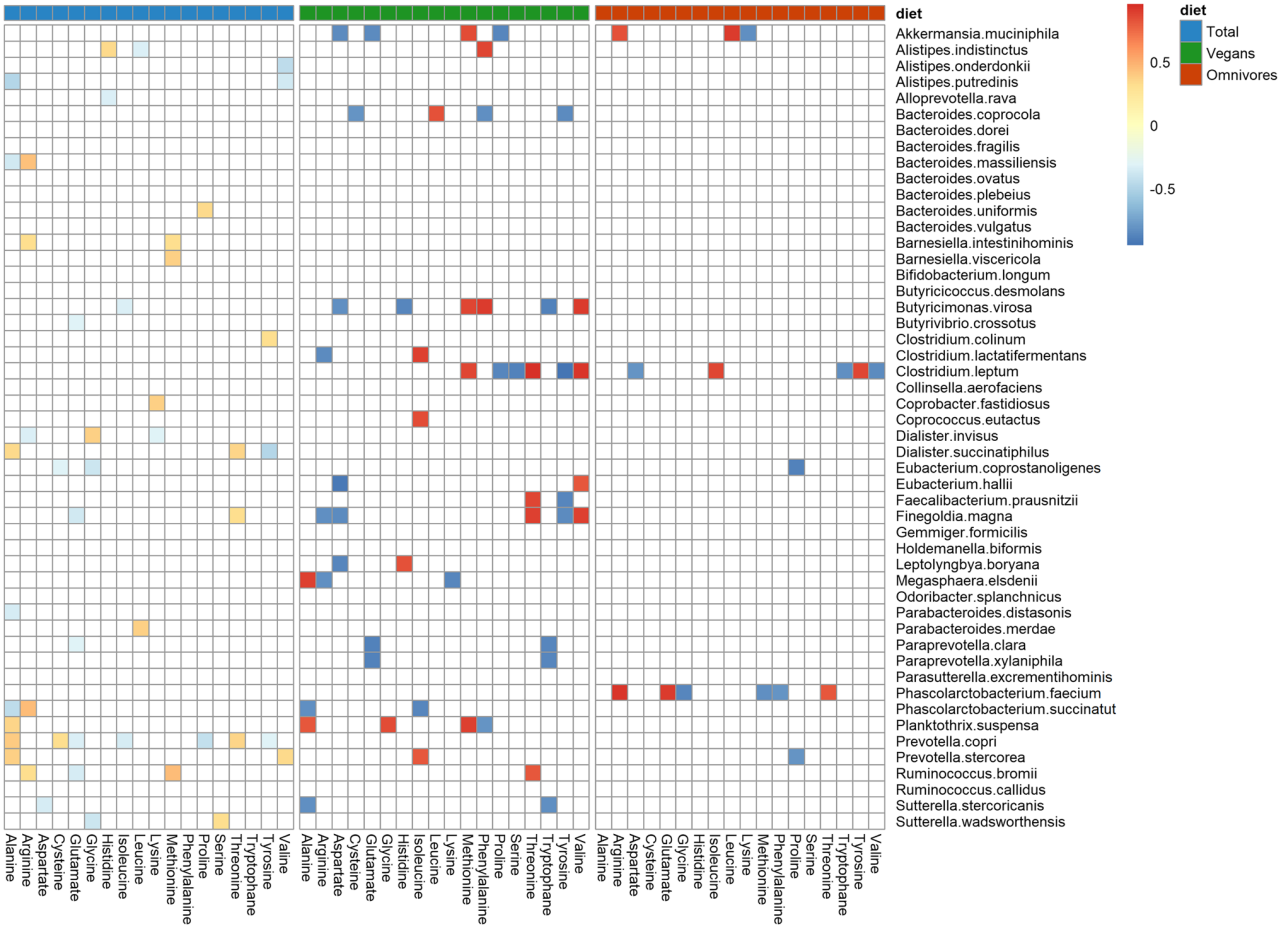
Partial Spearman correlation between dietary amino acids intake and gut bacterial species. Partial correlations were adjusted for age, sex, body mass index, physical activity, smoking status, alcohol consumption, amino acid supplementation, antibiotics, under-reporting, faecal pH and intake of fibre, carbohydrate and fat. Data of the 36 vegans and 36 omnivores were analysed. The colours in the figure represent partial correlation coefficients from − 1 to 1. Shown are only correlation coefficients with uncorrected *p* values < 0.05

The Spearman partial correlation pattern between the dietary intake of AAs and the intestinal bacteria differed significantly between participants with a vegan and an omnivorous diet across all taxonomic levels considering, among others, dietary fibre, fat and carbohydrate intake as confounder (Fig. 2, Supplemental Figs. S1–S4).

At species level, the dietary intake of AAs was correlated with 24 species in the combined total sample, but mostly with other species and weaker correlation coefficients (range: *r*_negative_: − 0.31 to − 0.48; *r*_positive_: 0.31–0.46) than in the two separately analysed dietary groups. In vegans, the dietary intake of individual AAs was significantly correlated (Fig. 2) with 19 species (range: –_negative_: − 0.81 to − 0.95; *r*_positive_: 0.81 to 0.96) and with five species in omnivores (range: *r*_negative_: − 0.81 to − 0.87; *r*_positive_: 0.82 to 0.93). The most abundant species in vegans (Supplemental Table S8, Fig. 2), with significant tested correlation coefficients, were *Faecalibacterium prausnitzii* (median number of reads: 968.5 (566.5–1774.5)), *Leptolyngbya boryana* (median number of reads: 92.0 (47.0–149.5)) and *Finegoldia magna* (median number of reads: 43.5 (33.0–63.0)). In vegans and omnivores (Fig. 2), the species *Akkermansia muciniphila, Clostridium leptum* and *Prevotella stercorea* were correlated with the dietary intake of some AAs, but mostly with different AAs in the respective dietary groups. In vegans (Fig. 2, Supplemental Fig. S1–S4), the intake of arginine, aspartate, glutamate, proline, tryptophan and tyrosine were mostly negatively correlated with bacterial abundance on all taxonomic levels. In contrast, isoleucine, methionine, threonine and valine were mostly positively correlated with bacterial abundance on all taxonomic levels in vegans. A few inconsistent correlation, pointing into both directions, was observed for histidine, cysteine, leucine, lysine and phenylalanine.

At genus level (Supplemental Fig. S4, Table S7), *Faecalibacterium* (median number of reads: 969 (567–178)), *Lachnoclostridium* (median number of reads: 973 (474–129)), *Ruminococcus* (median number of reads: 245 (123–394)), *Oscillibacter* (median number of reads: 224 (162–378)) and *Barnesiella* (median number of reads: 223 (22.5–829)) were the most abundant genera that showed a correlation with dietary intake of several AAs in vegans. In both dietary groups *Bacteroides* and *Alistipes,* known to be proteolytic fermenters, were not correlated with dietary intake of AAs at genus level.

## Discussion

The present study expands our knowledge of the AA status in vegans and observed an up to 57% lower dietary intake of nine AAs (seven essential AAs) for a vegan diet compared to an omnivorous diet. However, a comparison with the EAR of WHO [5] indicates that the vegans consumed sufficient amounts of essential AAs. Despite different intake levels, there were hardly any difference in plasma concentrations of AAs for the two dietary groups. Interestingly, the correlation analyses suggest that the different dietary intakes of AAs are associated with altered gut microbiota composition in vegans and omnivores.

The present study found no evidence that a vegan diet is generally associated with an inadequate dietary intake of total protein or essential AAs. The findings suggest a low proportion of vegans (1 out of 32 non-under-reporters) with a potential insufficient dietary intake of total protein and lysine. In addition, three of four potential under-reporters showed insufficient intake of some essential AAs. Three-day weighed food protocol or food frequency questionnaires (FFQs) are well established methods to assess diet and to classify participants according to amount of dietary intake [6]. Nevertheless, due to imprecise self-recorded information, they also bare the risk to underestimate and overestimate dietary intake resulting in uncertainties at the extreme range of protein and AA intake [6]. Moreover, in the present study, the three-day weighed food protocols could not be corrected for usual intake, because an FFQ which appropriately considered vegan foods was not available. For these reasons, under-reporters were excluded when the prevalence of insufficient intake was estimated. Another point to be mentioned is that all vegans showed a sufficient total intake of the sulphur-containing AAs (cysteine and methionine). But when looking at intake of methionine alone, six vegans, who were not under-reporters, showed an intake below the EAR (data not shown). However, from the dietary perspective, methionine should not be considered individually, but always together with cysteine, since cysteine has a sparing effect for methionine [5, 30, 31]. Nevertheless, the strength of the sparing effect of cysteine on methionine is still scientifically discussed [31].

Previous studies have reported lower total protein intake [6–9] in vegans compared to omnivores, but EPIC-Oxford was the only study which also investigated the AA status in detail, to date [7]. Findings in EPIC-Oxford and in the present study are largely consistent, even if different dietary assessment tools were used. In the EPIC-Oxford study [7], a FFQ was used that was not adapted to assess a vegan diet. Therefore, the dietary intake of AAs coming from vegan foods may have been underestimated [7]. In contrast, the present study used a three-day weighed food protocol, which also enabled the recording of vegan food consumption. This lowers the likelihood of underestimating the dietary intake of AAs coming from vegan foods. In addition, the present study weighted dietary intake according to body weight, which was not applied in EPIC-Oxford [7].

The present study also showed that the lower dietary intake of AAs in vegans was hardly reflected in the plasma concentrations of free AAs. Compared to omnivores, vegans showed lower plasma concentrations for lysine only and even higher concentrations for glutamine and glycine. This roughly corresponds with the EPIC-Oxford findings [7]. However, vegans in EPIC-Oxford showed also a trend towards lower methionine and tryptophan and higher alanine plasma concentrations than omnivores. However, in the present study, all participants fasted overnight before the blood sampling, while EPIC-Oxford also included participants with non-fasted blood samples [7]. This may explain the generally higher plasma concentrations of AAs found in EPIC-Oxford as compared to the present findings. Nevertheless, comparable plasma concentrations of AAs for vegans and omnivores, despite lower dietary intake of AAs among vegans, are not surprising, because free AA levels in blood are subject to homeostatic regulation [32, 33]. Despite homeostatic regulation, lysine plasma concentration was lower among vegans, which thus may reflect a permanently long-term reduced dietary intake of lysine in vegans compared to omnivores. While lysine is typically low in cereal-based products, plants often have high levels of glycine and glutamate [34], which may explain the higher concentrations of the two AAs among vegans.

Another interesting finding from the present study was that a vegan diet with lower dietary intake of AAs appears to be related to the bacterial gut microbiota. Notably, a previous analysis revealed only slight differences in microbiota diversity between the two dietary groups analysed here [26], despite the long-term veganism of 4.8 years (median) and the lower protein and higher fibre intake among vegans of the present study. To date, the effects of different dietary intake of proteins and individual AAs on bacterial gut microbiota have been poorly investigated. Nevertheless, the present findings are supported by some previous studies. It is assumed that the amount of proteins that enter the distal colon is a function of the amount of dietary protein intake and the absorption by enterocytes and microbes, as well as the transit time through the upper gastrointestinal tract [12, 35]. It has been reported that 3–12 g per day of the ingested proteins enters the distal colon [10] and is thus available for bacterial proteolytic fermentation. Consequently, the available AA substrate for bacterial proteolytic fermentation may differ in vegans and omnivores. Indeed, studies found microbiota composition to be modulated in response to altered dietary protein levels [36–38]. Moreover, higher levels of microbial protein hydrolysing enzymes and methionine transport systems [39], and upregulation of microbial valine, leucine and isoleucine degradation pathways [40] have been reported for vegetarians and vegans compared to omnivores. Functional annotations in mouse models also revealed that metabolic pathways related to AAs account for 16% of microbiota reactions and thus play an important role for microbiota [41].

Even though the complex relationship between dietary intake of AAs and gut bacterial microbiota is poorly understood, evidence suggests that a high animal-based protein diet may promote the growth of potential harmful gut bacteria from the genera *Bacteroides*, *Alistipes* and *Ruminococcus,* which are also associated with a higher risk of inflammatory bowel diseases and cardiovascular diseases [42]. In contrast, intake of proteins of plant origin is associated with a higher abundance of rather protective bacteria from the genera *Bifidobacterium* and *Lactobacillus* [42]. However, in the present study only *Ruminococcus* was correlated with dietary AA intake in vegans, while *Bacteroides*, *Alistipes, Bifidobacterium* and *Lactobacillus* showed no correlation in either dietary group. Association with health outcomes have also been found for some of the species (e.g. *A. muciniphila*, *C. leptum*, *F. prausnitzii* and *F. magna*) that showed correlations with dietary AA intake. For example, dietary interventions studies in mice and humans have shown that a higher abundance of *A. muciniphila*, a mucus colonizer, is associated with a lower risk of metabolic syndrome and intestinal inflammation [43]. Patients with inflammatory bowel diseases [44] showed a lower abundance of *C. leptum* and *F. prausnitzii*. In contrast, *F. magna* appears to have adverse health effects because it has the potential to induce inflammation [45]. Of these health-associated species, *F. prausnitzii* was the most abundant species in the present study, with a trend for higher abundance among vegans. In view of these previous findings, the observed associations of lower AA intake with certain health-associated microbiota due to a vegan diet suggest that this topic may be of particular relevance with regard to public health.

### Strengths and limitations

The strength of the present study is that both dietary groups have similar characteristics in lifestyle and anthropometric variables, and that comprehensive standardized data were collected, including the collection of blood, diet and faecal samples. The usage of a three-day weighed food protocol allowed the detailed food documentation, including vegan foods. This method can be considered as more reasonable to estimate the food intake in vegans than all currently in German available FFQ which did not include vegan food groups appropriately so far. However, it has to be noted that the AA content of a minor portion of the consumed vegan food could not be considered due to missing entries in the German Nutrient database. Unfortunately, the exact ingredients and thus the protein sources of these foods are not known, which prevented an estimate of the individual values of AAs by imputation. The strict inclusion of participants that followed their diet for at least one year (4.8-year median duration of veganism) is another strength. Accordingly, an adherence to a vegan diet and microbiota composition which is well adapted to the respective diet form could be assumed. In addition, the sample processing from excretion to freezing of faecal samples was carried out quickly, which counteracted possible degradation processes. Moreover, all participants fasted overnight before the blood sampling so that the AA plasma concentrations were not influenced by recent food consumption. The likelihood of confounding was reduced by matching the study groups for age and sex, and by adjusting microbiota analysis for AA supplements, antibiotics and macronutrients. In addition, a previous sensitivity analysis revealed that microbiota composition was not influenced by intake of antibiotics in the study [23].

Despite these strengths, the scientific significance of the present study is limited by the small sample size, resulting in lower statistical power. Therefore, uncorrected *p* values were reported for the partial Spearman correlation analyses. However, these findings may represent trends and serve as valuable information. Larger studies are needed to confirm these findings. Nonetheless, the obtained findings correspond with the findings of previous studies, and even expand current evidence regarding AA status and alterations of AAs with microbiota composition by a vegan diet. Due to the cross-sectional design, the change in AA status and gut microbiota composition over time could not be considered. Hence, no inference about causality can be made. The inclusion of only healthy, middle-aged participants limits the transferability to the general population. Disadvantages of a three-day weighed food protocol are that it also harbours the risk of reporting bias [46] due to self-reports, and that it covers only a small timeframe. In the present study, the three-day weighed food protocol could not be corrected for usual intake because no FFQ was available that appropriately considered also vegan food groups. Therefore, the likelihood exists that the proportion of protein and AAs intakes of consumers has been underestimated, which is a particular problem when using EAR as a cut-off. In order to take this point more into account, we have excluded under-reporters from the analysis with EAR.

## Conclusions

In conclusion, the present study improved the scientific evidence to evaluate whether a vegan diet is associated with a sufficient dietary intake of AAs. However, the present findings must be interpreted with caution as they are based on short-term dietary intake data without considering usual intake. Although uncertainties remain about underestimating or overestimation of AA intake, it appears that the dietary intake of AAs in the investigated vegans was in line with the EAR of WHO. This is supported by the relatively comparable plasma concentrations of AAs between vegans and omnivores. Furthermore, the present findings suggest that different AA intake in vegans and omnivores may be associated with changes in gut microbiota ecology. Considering the potential protective or harmful effects of the identified gut bacteria on host health, and that proteolytic fermentation is a highly networked process, more information is needed. Therefore, the reproducibility and validity of the findings should be investigated in further studies comprising larger study populations and considering usual intake by the usage of FFQs which are adapted to vegan diets.

## Supplementary Information

Below is the link to the electronic supplementary material.

Supplementary file1 (DOCX 1345 KB)

## Data Availability

The data sets generated and/or analysed during the current Risks and Benefits of a Vegan Diet study are not publicly available due to provisions of the data protection regulations.

## Code availability

Data are available upon request.
