# Supplementary material for: Amino acid intake and plasma concentrations and their interplay with gut microbiota in vegans and omnivores in Germany
Source: Eur J Nutr. 2022 Jan 16;61(4):2103–14. doi: 10.1007/s00394-021-02790-y (PMC9106628; doi:10.1007/s00394-021-02790-y)
Supplement: Supplementary file 1 — Supplementary file1 (DOCX 1345 KB) [file 394_2021_2790_MOESM1_ESM.docx]

**Supplementary Appendix**

**Amino acid intake and plasma concentrations and their interplay with gut microbiota in vegans and omnivores in Germany**

Stefan Dietrich^1^, Iris Trefflich^1^, Per Magne Ueland^2^, Juliane Menzel^1,3^, Katharina J. Penczynski^1^, Klaus Abraham^1^, Cornelia Weikert^1^

1 German Federal Institute for Risk Assessment, Department of Food Safety, Berlin, Germany.

2 BEVITAL AS, Bergen, Norway

3 Institute of Social Medicine, Epidemiology and Health Economics, Charité – Universitätsmedizin Berlin, corporate member of Freie Universität Berlin and Humboldt-Universität zu Berlin, Berlin, Germany

**Table S1:** Differences across tertiles of protein intake in g/day per kg body weight

|  | **Vegans**^a^ (n=36) | | | | **Omnivores**^a^ (n=36) | | | |
| --- | --- | --- | --- | --- | --- | --- | --- | --- |
|  | **1.Tertile** | **2. Tertile** | **3. Tertile** | **p-value**^b^ | **1. Tertile** | **2. Tertile** | **3. Tertile** | **p-value**^b^ |
| Protein intake (g/day/kg bodyweight) | 0.73 (0.63-0.84) | 1.04 (0.94-1.15) | 1.58 (1.48-1.69) | **<0.0001** | 0.95 (0.63-1.27) | 1.20 (0.89-1.52) | 1.86 (1.54-2.18) | **0.0002** |
| Fibre (g/kg/day) | 0.50 (0.40-0.60) | 0.65 (0.55-0.74) | 0.88 (0.55-0.74) | **<0.0001** | 0.29 (0.19-0.39) | 0.35 (0.25-0.45) | 0.43 (0.33-0.53) | 0.05 |
| Fat (g/kg/day) | 1.02 (0.77-1.28) | 1.42 (1.17-1.67) | 1.41 (1.15-1.66) | 0.80 | 1.30 (0.92-1.67) | 1.64 (1.26-2.01) | 1.88 (1.50-2.25) | **0.03** |
| Carbohydrate (g/kg/day) | 3.66 (3.00-4.32) | 4.23 (3.60-4.88) | 4.51 (3.85-5.17) | 0.07 | 3.33 (2.00-4.64) | 3.41 (2.09-4.72) | 4.14 (2.83-5.46) | 0.38 |
| Ammonium (µmol/g) | 24.1 (16.6-31.5) | 28.0 (20.6-35.5) | 31.3 (23.9-38.7) | 0.17 | 41.9 (31.2-52.6) | 28.7 (18.0-39-4) | 37.1 (26.4-47.8) | 0.52 |
| Muscle mass (%) | 34.1 (31.0-37.3) | 34.2 (31.0-36.6) | 33.4 (30.3-36.6) | 0.75 | 32.5 (29.4-35.6) | 33.5 (30.6-36.6) | 34.4 (31.2-37.5) | 0.40 |
| Bodyfat mass (%) | 24.6 (19.9-29.3) | 22.1 (17.4-26.7) | 25.7 (21.1-30.4) | 0.73 | 27.9 (23.3-32.5) | 26.5 (21.9-31.0) | 24.2 (19.6-28.7) | 0.25 |
| Physical activity (h/week) | 2.50 (1.04-3.95) | 2.20 (0.74-3.65) | 3.73 (2.27-5.18) | 0.23 | 2.47 (1.33-3.61) | 2.50 (1.33-3.61) | 3.52 (2.38-4.66) | 0.19 |
| Faeces pH | 6.54 (6.26-6.82) | 6.23 (5.96-6.51) | 6.44 (6.16-6.72) | 0.60 | 6.55 (6.29-6.81) | 6.86 (6.60-7.11) | 6.78 (6.51-7.06) | 0.21 |

^a^ Data are reported as median (IQR).

^b^ Differences between tertiles were tested with Kruskal Wallis test

**Table S2:** Dietary intake of amino acid in the RBVD study after exclusion of under-reporter.

| Amino acids | Vegans^a^  (n=32) | Omnivores^a^  (n=34) | Median difference [%] | raw  p-value^b^ | corrected  p-value^b,c^ |  |
| --- | --- | --- | --- | --- | --- | --- |
| Essential and semi-essential amino acids [mg/d per kg body weight] | | | | | | |
| Histidine | 20.6 (18.9 - 32.7) | 32.0 (27.9 - 38.8) | -35.6 | <0.001 | **0.003** |  |
| Isoleucine | 40.0 (34.0 - 60.5) | 56.0 (47.6 - 65.3) | -28.6 | <0.001 | **0.02** |  |
| Leucine | 65.3 (56.0 - 97.9) | 95.5 (79.8 - 112) | -31.6 | <0.001 | **0.01** |  |
| Lysine | 41.8 (34.2 - 69.1) | 78.9 (63.2 - 96.9) | -47.0 | <0.001 | **<0.001** |  |
| Methionine | 12.9 (11.7 - 17.5) | 26.0 (22.4 - 31.3) | -50.4 | <0.001 | **<0.001** |  |
| Phenylalanine | 44.0 (37.4 - 66.5) | 54.7 (46.0 - 61.8) | -19.6 | 0.07 | 1 |  |
| Threonine | 33.1 (28.5 - 49.9) | 47.3 (40.3 - 57.5) | -30.0 | 0.001 | **0.02** |  |
| Tryptophan | 11.3 (9.8 - 15.5) | 14.3 (12.5 - 16.3) | -21.0 | 0.03 | 0.53 |  |
| Valine | 45.5 (40.2 - 69.7) | 67.7 (58.0 - 73.8) | -32.8 | <0.001 | **0.02** |  |
| Total | 310.5 (270.8 - 480.2) | 476.9 (401.1 - 547.7) | -34.9 | <0.001 | **0.007** |  |
| Non-essential amino acids [mg/d per kg body weight] | | | | | | |
| Alanine | 58.8 (42.9 - 71.4) | 58.7 (48.2 - 74.7) | +0.17 | 0.40 | 1 |  |
| Arginine | 64.8 (50.2 - 89.4) | 65.5 (51.9 - 76.6) | -1.1 | 0.67 | 1 |  |
| Aspartate/Asparagine | 94.0 (75.6 - 137.2) | 103.7 (85.0 - 123.8) | -9.4 | 0.38 | 1 |  |
| Cysteine | 16.6 (13.9 - 21.8) | 16.1 (13.8 - 18.3) | +3.1 | 0.61 | 1 |  |
| Glutamate/Glutamine | 220.8 (181.2 – 282.0) | 250.1 (228.0 - 295.5) | -11.7 | 0.08 | 1 |  |
| Glycine | 41.9 (36.4 - 58.9) | 45.6 (40.4 - 60.2) | -8.1 | 0.23 | 1 |  |
| Proline | 68.8 (55.5 - 87.1) | 92.1 (79.0 - 103.4) | -25.3 | <0.001 | **0.003** |  |
| Serine | 47.7 (41.4 – 68.0) | 61.4 (53.0 - 67.6) | -22.3 | 0.04 | 0.75 |  |
| Tyrosine | 28.5 (24.5 - 42.6) | 44.6 (35.8 - 50.5) | -36.1 | <0.001 | **0.001** |  |
| Total | 632.8 (552.3 - 856.5) | 732.3 (663.1 - 840.9) | -13.6 | 0.08 | 1 |  |

^a^ Data are reported as median (IQR).

^b^ Differences between groups were tested with Kruskal Wallis test.

^c^ Bonferroni correction was applied to correct p-values for multiple testing (raw p-value multiplied by 20)

**Table S3:** Essential amino acid status in four under-reporter with a vegan diet compared to EAR of WHO.

| Amino acids  [mg/kg body weight per day] | EAR of WHO^a^ | Dietary intake [mg/kg per day] | | | prevalence of inadequate intakes  [% (n)] |
| --- | --- | --- | --- | --- | --- |
|  |  | **min** | **median (IQR)** | **max** |  |
| Histidine | 10.0 | 9.9 | 12 (10.6 - 14.1) | 15.4 | 25 (1) |
| Isoleucine | 20.0 | 17.05 | 21.2 (18.5 - 25.1) | 27.8 | 25 (1) |
| Leucine | 39.0 | 29.5 | 34.8 (30.5 - 41.1) | 44.3 | 75 (3) |
| Lysine | 30.0 | 15.0 | 23.8 (19.4 - 27.9) | 31.9 | 75 (3) |
| SAA | 15.0 | 12.58 | 14.5 (13.1 - 17.6) | 19.89 | 50 (2) |
| AAA | 25.0 | 31.28 | 38.1 (32.6 - 45.9) | 49.55 | 0 (0) |
| Threonine | 15.0 | 14.2 | 17.6 (15.7 - 20.7) | 23.3 | 25 (1) |
| Tryptophan | 4.0 | 4.5 | 6.8 (5.5 - 7.9) | 8.7 | 0 (0) |
| Valine | 26.0 | 20.6 | 25.1 (22.6 - 28.4) | 31.1 | 75 (3) |
| Total Protein [g/kg body weight per day] | 0.66 | 0.47 | 0.5 (0.5 - 0.7) | 0.74 | 75 (3) |

^a^ as reported in chapter 8, Table 23 [5]

Abbreviations: AAA, aromatic amino acids (phenylalanine and tyrosine); EAR, estimated average requirements; SAA, sulphur amino acids

**Table S4:** Abundances of taxa at phylum level in vegans and omnivores.

| **Phylum** | **Vegans**^a^  **(n=36)** | **Omnivores**^a^  **(n=36)** | **p-value**^b^ |
| --- | --- | --- | --- |
| *Actinobacteria* | 347.5 (133 - 677.5) | 266 (143.5 - 555) | 0.73 |
| *Bacteroidetes* | 12127 (9552 - 16383) | 12958 (8857 - 16517) | 0.84 |
| *Cyanobacteria* | 153 (96 - 250.5) | 149.5 (96 - 200) | 0.77 |
| *Firmicutes* | 17357.5 (13550 - 22231.5) | 15603 (12928 - 18595.5) | 0.16 |
| *Proteobacteria* | 426.5 (204 - 875.5) | 544 (311 - 997.5) | 0.21 |
| *Synergistetes* | 2.5 (0.5 - 6.5) | 2 (1 - 7) | 0.88 |
| *Tenericutes* | 13.5 (0 - 60) | 1 (0 - 13.5) | **0.02** |
| *Verrucomicrobia* | 32.5 (3 - 697) | 40 (1.5 - 566) | 0.61 |

^a^ Data are reported as median (IQR) abundances of reads resulting from 16S rRNA sequencing.

^b^ Differences between groups were tested with Kruskal Wallis test.

**Table S5:** Abundances of taxa at class level in vegans and omnivores.

| **Class** | **Vegans**^a^  **(n=36)** | **Omnivores**^a^  **(n=36)** | **p-value**^b^ |
| --- | --- | --- | --- |
| *Actinobacteria* | 193.5 (76 - 484) | 170 (54.5 - 412.5) | 0.64 |
| *Alphaproteobacteria* | 36.5 (22 - 78) | 37.5 (15 - 118.5) | 0.99 |
| *Bacilli* | 118.5 (72 - 196) | 93 (66.5 - 157) | 0.21 |
| *Bacteroidia* | 12068 (9521 - 16213) | 12887 (8779.5 - 16463.5) | 0.83 |
| *Betaproteobacteria* | 222 (84.5 - 492.5) | 183.5 (64 - 524.5) | 0.97 |
| *Clostridia* | 14588.5 (10915 - 19035) | 12609.5 (10544.5 - 16816.5) | 0.17 |
| *Coriobacteriia* | 64.5 (21.5 - 134) | 99.5 (38.5 - 163.5) | 0.16 |
| *Deltaproteobacteria* | 48 (21.5 - 88.5) | 58 (21.5 - 165) | 0.37 |
| *Erysipelotrichia* | 61.5 (35.5 - 125) | 77 (44.5 - 175) | 0.36 |
| *Gammaproteobacteria* | 27 (20 - 49.5) | 55.5 (19.5 - 149.5) | 0.18 |
| *Mollicutes* | 13.5 (0 - 60) | 1 (0 - 13.5) | **0.02** |
| *Negativicutes* | 1140 (674.5 - 2327.5) | 1244 (791.5 - 1779) | 0.99 |
| *Sphingobacteriia* | 1 (0 - 2.5) | 2 (1 - 4.5) | 0.06 |
| *Synergistia* | 2.5 (0.5 - 6.5) | 2 (1 - 7) | 0.88 |
| *Tissierellia* | 47 (37 - 68.5) | 47 (34.5 - 58) | 0.73 |
| *Verrucomicrobiae* | 32.5 (3 - 697) | 40 (1.5 - 566) | 0.49 |

^a^ Data are reported as median (IQR) abundances of reads resulting from 16S rRNA sequencing.

^b^ Differences between groups were tested with Kruskal Wallis test.

**Table S6:** Abundances of taxa at family level in vegans and omnivores

| **Family** | **Vegans**^a^  **(n=36)** | **Omnivores**^a^  **(n=36)** | **p-value**^b^ |
| --- | --- | --- | --- |
| *Acidaminococcaceae* | 116.5 (2 - 572) | 233 (2 - 867) | 0.59 |
| *Akkermansiaceae* | 31 (3 - 667) | 37 (1 - 540.5) | 0.43 |
| *Atopobiaceae* | 1 (0 - 5.5) | 1 (0 - 16) | 0.66 |
| *Bacteroidaceae* | 6352 (1892.5 - 8668.5) | 6094 (3097 - 11394.5) | 0.37 |
| *Bifidobacteriaceae* | 150.5 (58.5 - 367) | 92 (29 - 351.5) | 0.39 |
| *Clostridiaceae* | 178 (108 - 275.5) | 100.5 (55 - 178) | **0.01** |
| *Coriobacteriaceae* | 28 (6 - 61) | 34.5 (16 - 74.5) | 0.37 |
| *Desulfovibrionaceae* | 42.5 (15.5 - 80) | 48.5 (15 - 157.5) | 0.40 |
| *Eggerthellaceae* | 17 (8 - 36) | 29.5 (10.5 - 76.5) | **0.03** |
| *Enterobacteriaceae* | 11 (2 - 21.5) | 19.5 (5.5 - 79) | 0.07 |
| *Erysipelotrichaceae* | 61.5 (35.5 - 125) | 77 (44.5 - 175) | 0.36 |
| *Eubacteriaceae* | 113.5 (61 - 251) | 96 (64 - 202.5) | 0.85 |
| *Lachnospiraceae* | 4502 (2892.5 - 6178) | 3590.5 (2583.5 - 5151) | 0.24 |
| *Lactobacillaceae* | 8.5 (5 - 33.5) | 5.5 (3 - 11.5) | 0.06 |
| *Leptolyngbyaceae* | 92 (47 - 149.5) | 85.5 (59.5 - 141.5) | 0.82 |
| *Microcoleaceae* | 40 (18 - 71.5) | 31.5 (16 - 77.5) | 0.77 |
| *Odoribacteraceae* | 94.5 (32 - 176.5) | 80.5 (28.5 - 151.5) | 0.72 |
| *Oscillospiraceae* | 224 (162 - 377.5) | 273 (178.5 - 360.5) | 0.91 |
| *Pasteurellaceae* | 4.5 (1 - 16) | 1 (0 - 7) | 0.04 |
| *Peptoniphilaceae* | 44.5 (33.5 - 65) | 46.5 (32.5 - 55) | 0.70 |
| *Peptostreptococcaceae* | 27 (18.5 - 41) | 28.5 (16 - 43.5) | 0.97 |
| *Porphyromonadaceae* | 1181 (451 - 1840) | 1065.5 (598.5 - 2174.5) | 0.65 |
| *Prevotellaceae* | 514 (35 - 7107.5) | 228.5 (27 - 3828.5) | 0.35 |
| *Rhodospirillaceae* | 1 (0 - 7) | 1 (0 - 5) | 0.89 |
| *Rikenellaceae* | 639.5 (260.5 - 1278.5) | 754 (514 - 1721.5) | 0.31 |
| *Ruminococcaceae* | 3720 (2270 - 5594) | 3880 (2791.5 - 5492) | 0.83 |
| *Selenomonadaceae* | 4 (1 - 6) | 4 (1 - 7.5) | 0.97 |
| *Sphingobacteriaceae* | 1 (0 - 2.5) | 2 (1 - 4.5) | 0.06 |
| *Spiroplasmataceae* | 6.5 (0 - 31.5) | 0 (0 - 12) | 0.05 |
| *Streptococcaceae* | 8 (5 - 45.5) | 21 (8 - 41) | 0.28 |
| *Sutterellaceae* | 106.5 (42.5 - 274.5) | 134 (37.5 - 416) | 0.81 |
| *Synergistaceae* | 2.5 (0.5 - 6.5) | 2 (1 - 7) | 0.88 |
| *Veillonellaceae* | 555 (25.5 - 1870) | 360 (18 - 1405.5) | 0.60 |
| *Verrucomicrobiaceae* | 0.5 (0 - 21.5) | 1 (0 - 17) | 0.51 |

^a^ Data are reported as median (IQR) abundances of reads resulting from 16S rRNA sequencing.

^b^ Differences between groups were tested with Kruskal Wallis test.

**Table S7:** Abundances of taxa at genus level in vegans and omnivores

| **Genus** | **Vegans**^a^  **(n=36)** | **Omnivores**^a^  **(n=36)** | **p-value**^b^ |
| --- | --- | --- | --- |
| *Acidaminococcus* | 2 (0 - 8) | 2.5 (0 - 7) | 0.84 |
| *Akkermansia* | 31 (3 - 667) | 37 (1 - 540.5) | 0.43 |
| *Alistipes* | 638.5 (259.5 - 1277.5) | 752.5 (512.5 - 1718.5) | 0.31 |
| *Alloprevotella* | 1 (0 - 2) | 0.5 (0 - 2) | 0.80 |
| *Anaerofilum* | 25 (15 - 57) | 39.5 (21.5 - 58.5) | 0.34 |
| *Anaerostipes* | 19 (6.5 - 37.5) | 17 (9 - 32) | 0.96 |
| *Bacteroides* | 6352 (1892.5 - 8668.5) | 6094 (3096.5 - 11394.5) | 0.37 |
| *Barnesiella* | 223 (22.5 - 829) | 327.5 (153 - 671.5) | 0.42 |
| *Bifidobacterium* | 147 (57.5 - 364.5) | 91 (27 - 344.5) | 0.40 |
| *Blautia* | 83.5 (41 - 146) | 101.5 (53.5 - 152) | 0.54 |
| *Butyricicoccus* | 114.5 (52 - 211.5) | 45.5 (26 - 93.5) | **0.002** |
| *Butyricimonas* | 25.5 (1.5 - 73.5) | 19 (8 - 56.5) | 0.98 |
| *Butyrivibrio* | 1 (0 - 3) | 1.5 (0.5 - 3) | 0.47 |
| *Clostridium* | 29.5 (18 - 58.5) | 22.5 (11.5 - 44.5) | 0.25 |
| *Collinsella* | 22.5 (5.5 - 47) | 32 (11.5 - 57.5) | 0.42 |
| *Coprobacter* | 3 (0.5 - 41) | 10.5 (1 - 34) | 0.54 |
| *Coprococcus* | 49 (12.5 - 231.5) | 38 (5 - 183.5) | 0.33 |
| *Desulfovibrio* | 39.5 (15 - 79.5) | 47.5 (14.5 - 155.5) | 0.40 |
| *Dialister* | 392 (6.5 - 1190.5) | 252 (6.5 - 1363) | 0.82 |
| *Erysipelatoclostridium* | 13 (7 - 38) | 29.5 (7 - 44) | 0.33 |
| *Eubacterium* | 113 (59 - 240) | 92.5 (62.5 - 202.5) | 0.85 |
| *Faecalibacterium* | 968.5 (566.5 - 1774.5) | 637 (439.5 - 1165) | 0.08 |
| *Finegoldia* | 43.5 (33 - 63) | 44 (31 - 53) | 0.67 |
| *Gemmiger* | 14.5 (5.5 - 36) | 34 (17.5 - 54.5) | **0.038** |
| *Haemophilus* | 2.5 (1 - 12) | 0 (0 - 4) | **0.027** |
| *Holdemanella* | 1 (0 - 33) | 0 (0 - 38.5) | 0.64 |
| *Lachnoclostridium* | 973 (473.5 - 1287.5) | 1091 (442.5 - 1950.5) | 0.44 |
| *Lactobacillus* | 8 (4 - 33.5) | 5 (2.5 - 10.5) | 0.051 |
| *Leptolyngbya* | 92 (47 - 149.5) | 85.5 (59.5 - 141.5) | 0.82 |
| *Megasphaera* | 2 (0.5 - 5.5) | 1 (0 - 4) | 0.28 |
| *Odoribacter* | 62 (22 - 116) | 46.5 (19 - 110) | 0.82 |
| *Oscillibacter* | 224 (162 - 377.5) | 273 (178.5 - 360.5) | 0.91 |
| *Parabacteroides* | 454.5 (247.5 - 759) | 550.5 (264 - 1230.5) | 0.29 |
| *Paraprevotella* | 22 (0 - 283.5) | 3 (1 - 145) | 0.51 |
| *Parasutterella* | 6 (1 - 15) | 13 (3.5 - 96.5) | **0.045** |
| *Phascolarctobacterium* | 72.5 (1.5 - 529) | 21.5 (1 - 690) | 0.95 |
| *Planktothrix* | 35.5 (18 - 59) | 26.5 (16 - 77.5) | 0.65 |
| *Prevotella* | 157.5 (26 - 6581.5) | 56.5 (25.5 - 2488.5) | 0.40 |
| *Roseburia* | 88.5 (46 - 234) | 77.5 (39.5 - 168) | 0.28 |
| *Ruminiclostridium* | 113 (38 - 197.5) | 96.5 (64 - 134.5) | 0.44 |
| *Ruminococcus* | 245 (122.5 - 393.5) | 325.5 (96 - 626) | 0.53 |
| *Spiroplasma* | 6.5 (0 - 31.5) | 0 (0 - 12) | **0.047** |
| *Streptococcus* | 8 (5 - 37.5) | 16.5 (6.5 - 41) | 0.32 |
| *Sutterella* | 76 (22.5 - 244.5) | 49 (3 - 200.5) | 0.33 |
| *Tyzzerella* | 21 (11 - 45) | 15 (5.5 - 25) | 0.11 |
| *Veillonella* | 8 (4 - 21.5) | 7 (4 - 14) | 0.49 |

^a^ Data are reported as median (IQR) abundances of reads resulting from 16S rRNA sequencing.

^b^ Differences between groups were tested with Kruskal Wallis test.

**Table S8:** Abundances of taxa at species level in vegans and omnivores

| **Species** | **Vegans**^a^  **(n=36)** | **Omnivores**^a^  **(n=36)** | **p-value** |
| --- | --- | --- | --- |
| *Akkermansia.muciniphila* | 31 (3 - 667) | 37 (1 - 540.5) | 0.426 |
| *Alistipes.indistinctus* | 17.5 (1 - 76) | 27 (4 - 64.5) | 0.257 |
| *Alistipes.onderdonkii* | 1 (0 - 12.5) | 4 (1 - 30) | 0.177 |
| *Alistipes.putredinis* | 9 (1.5 - 33) | 22 (2 - 37) | 0.175 |
| *Alloprevotella.rava* | 1 (0 - 1) | 0 (0 - 1.5) | 0.619 |
| *Bacteroides.coprocola* | 1 (0 - 6) | 1 (0 - 2) | 0.132 |
| *Bacteroides.dorei* | 10.5 (2 - 43.5) | 17 (7 - 37.5) | 0.398 |
| *Bacteroides.fragilis* | 23.5 (7 - 85.5) | 30 (15 - 85.5) | 0.237 |
| *Bacteroides.massiliensis* | 4 (2 - 132.5) | 5 (2 - 165) | 0.599 |
| *Bacteroides.ovatus* | 23.5 (7 - 58) | 23 (10 - 69.5) | 0.562 |
| *Bacteroides.plebeius* | 1 (0 - 6.5) | 0 (0 - 1.5) | 0.121 |
| *Bacteroides.uniformis* | 12.5 (1.5 - 32.5) | 35 (14 - 71) | **0.004** |
| *Bacteroides.vulgatus* | 9.5 (2.5 - 20) | 17.5 (6 - 34.5) | 0.070 |
| *Barnesiella.intestinihominis* | 8.5 (1 - 27.5) | 15 (3.5 - 30) | 0.190 |
| *Barnesiella.viscericola* | 3 (0.5 - 6.5) | 2 (0.5 - 5.5) | 0.986 |
| *Bifidobacterium.longum* | 4.5 (1 - 15.5) | 6 (0.5 - 17) | 0.712 |
| *Butyricicoccus.desmolans* | 5.5 (2 - 35.5) | 2 (1 - 18) | **0.049** |
| *Butyricimonas.virosa* | 0 (0 - 2) | 0.5 (0 - 5) | 0.134 |
| *Butyrivibrio.crossotus* | 0 (0 - 1) | 0.5 (0 - 1) | 0.248 |
| *Clostridium.colinum* | 3 (0 - 12) | 0 (0 - 1) | **0.004** |
| *Clostridium.lactatifermentans* | 1 (0 - 3) | 2 (0 - 3.5) | 0.321 |
| *Clostridium.leptum* | 7 (2.5 - 14.5) | 9.5 (5 - 15) | 0.245 |
| *Collinsella.aerofaciens* | 14 (3.5 - 31) | 22 (4.5 - 43.5) | 0.286 |
| *Coprobacter.fastidiosus* | 3 (0.5 - 41) | 10.5 (1 - 34) | 0.540 |
| *Coprococcus.eutactus* | 41.5 (2 - 226) | 31 (0 - 176.5) | 0.366 |
| *Dialister.invisus* | 2 (0 - 20.5) | 4.5 (2 - 393) | **0.043** |
| *Dialister.succinatiphilus* | 2.5 (0.5 - 81.5) | 0 (0 - 2.5) | **0.015** |
| *Eubacterium.coprostanoligenes* | 57 (18 - 229.5) | 70.5 (30.5 - 160) | 0.982 |
| *Eubacterium.hallii* | 6.5 (3 - 13.5) | 9.5 (5.5 - 14.5) | 0.160 |
| *Faecalibacterium.prausnitzii* | 968.5 (566.5 - 1774.5) | 637 (439.5 - 1165) | 0.077 |
| *Finegoldia.magna* | 43.5 (33 - 63) | 44 (31 - 53) | 0.673 |
| *Gemmiger.formicilis* | 14.5 (5.5 - 36) | 34 (17.5 - 54.5) | **0.038** |
| *Holdemanella.biformis* | 1 (0 - 33) | 0 (0 - 38.5) | 0.635 |
| *Leptolyngbya.boryana* | 92 (47 - 149.5) | 85.5 (59.5 - 141.5) | 0.822 |
| *Megasphaera.elsdenii* | 1.5 (0 - 5) | 1 (0 - 3.5) | 0.496 |
| *Odoribacter.splanchnicus* | 48 (19.5 - 110.5) | 46 (18 - 105) | 0.879 |
| *Parabacteroides.distasonis* | 191 (77 - 326.5) | 175 (87 - 518.5) | 0.669 |
| *Parabacteroides.merdae* | 15.5 (2.5 - 29.5) | 12.5 (1 - 63) | 0.721 |
| *Paraprevotella.clara* | 1 (0 - 258.5) | 1 (0 - 123.5) | 0.803 |
| *Paraprevotella.xylaniphila* | 0.5 (0 - 4) | 0 (0 - 1.5) | 0.207 |
| *Parasutterella.excrementihominis* | 6 (1 - 15) | 13 (3.5 - 96.5) | **0.043** |
| *Phascolarctobacterium.faecium* | 9.5 (1 - 484.5) | 3 (1 - 401) | 0.675 |
| *Phascolarctobacterium.succinatut* | 1 (0 - 3) | 2 (0 - 4.5) | 0.377 |
| *Planktothrix.suspensa* | 35.5 (18 - 59) | 26.5 (16 - 77.5) | 0.648 |
| *Prevotella.copri* | 19 (8.5 - 5250.5) | 19 (9 - 1640.5) | 0.765 |
| *Prevotella.stercorea* | 1 (0 - 6) | 1 (0 - 2.5) | 0.393 |
| *Ruminococcus.bromii* | 52.5 (2.5 - 114) | 64.5 (22 - 146.5) | 0.477 |
| *Ruminococcus.callidus* | 17.5 (0 - 35.5) | 5.5 (0 - 27.5) | 0.346 |
| *Sutterella.stercoricanis* | 1 (0 - 40.5) | 0 (0 - 4.5) | 0.106 |
| *Sutterella.wadsworthensis* | 26.5 (2.5 - 153) | 17 (1 - 76) | 0.312 |

^a^ Data are reported as median (IQR) abundances of reads resulting from 16S rRNA sequencing.

^b^ Differences between groups were tested with Kruskal Wallis test.

**Figure S1:** Spearman partial correlation of dietary amino acids intake with phyla


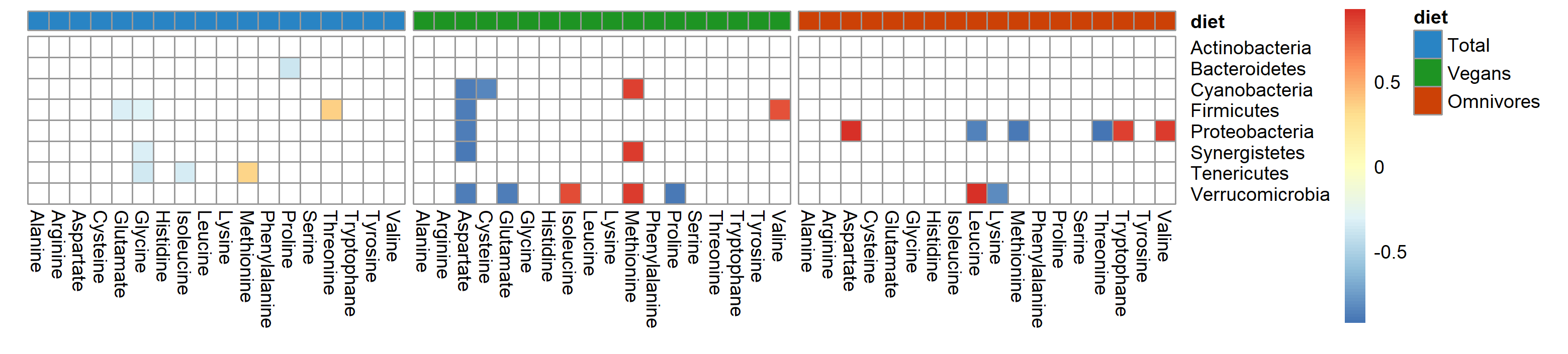


Partial correlations were adjusted for age, sex, body mass index, physical activity, smoking status, alcohol consumption, amino acid supplementation, antibiotics, under-reporting, faecal pH and intake of fibre, carbohydrate and fat. Data of the 36 vegans and 36 omnivores were analysed. The colours in the figure represent partial correlation coefficients from -1 to 1. Shown are only correlation coefficients with uncorrected P-values < 0.05.

**Figure S2:** Spearman partial correlation of dietary amino acids intake with classes


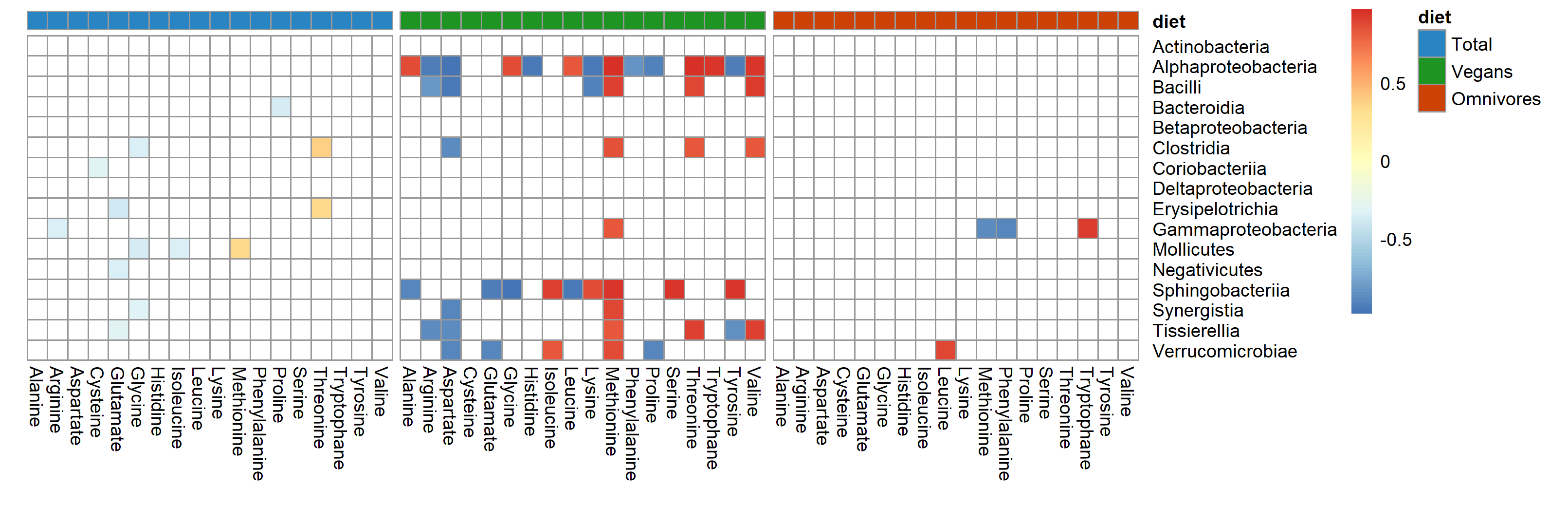


Partial correlations were adjusted for age, sex, body mass index, physical activity, smoking status, alcohol consumption, amino acid supplementation, antibiotics, under-reporting, faecal pH and intake of fibre, carbohydrate and fat. Data of the 36 vegans and 36 omnivores were analysed. The colours in the figure represent partial correlation coefficients from -1 to 1. Shown are only correlation coefficients with uncorrected P-values < 0.05.

**Figure S3:** Spearman partial correlation of dietary amino acids intake with families


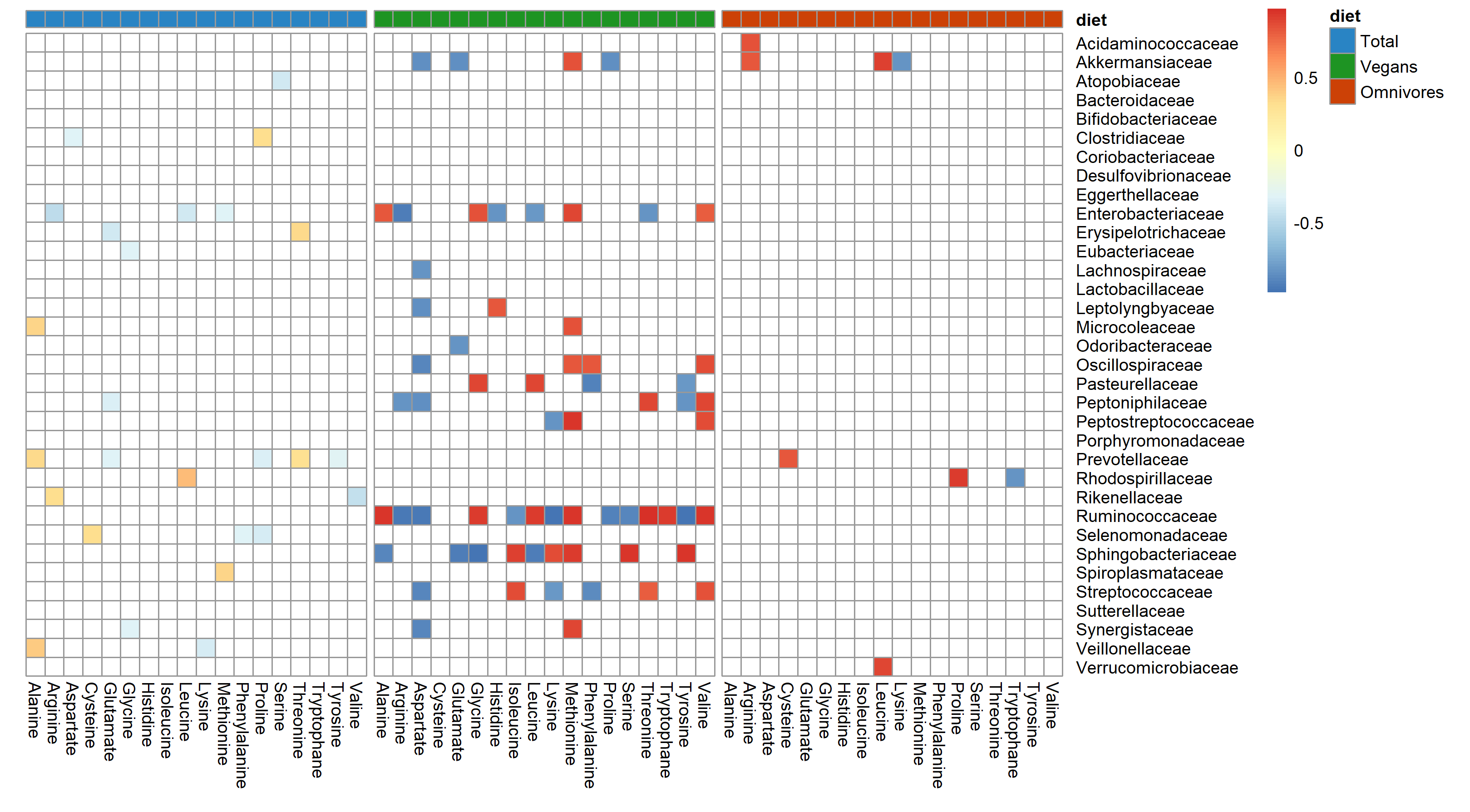


Partial correlations were adjusted for age, sex, body mass index, physical activity, smoking status, alcohol consumption, amino acid supplementation, antibiotics, under-reporting, faecal pH and intake of fibre, carbohydrate and fat. Data of the 36 vegans and 36 omnivores were analysed. The colours in the figure represent partial correlation coefficients from -1 to 1. Shown are only correlation coefficients with uncorrected P-values < 0.05.

**Figure S4:** Spearman partial correlation of dietary amino acids intake with genus


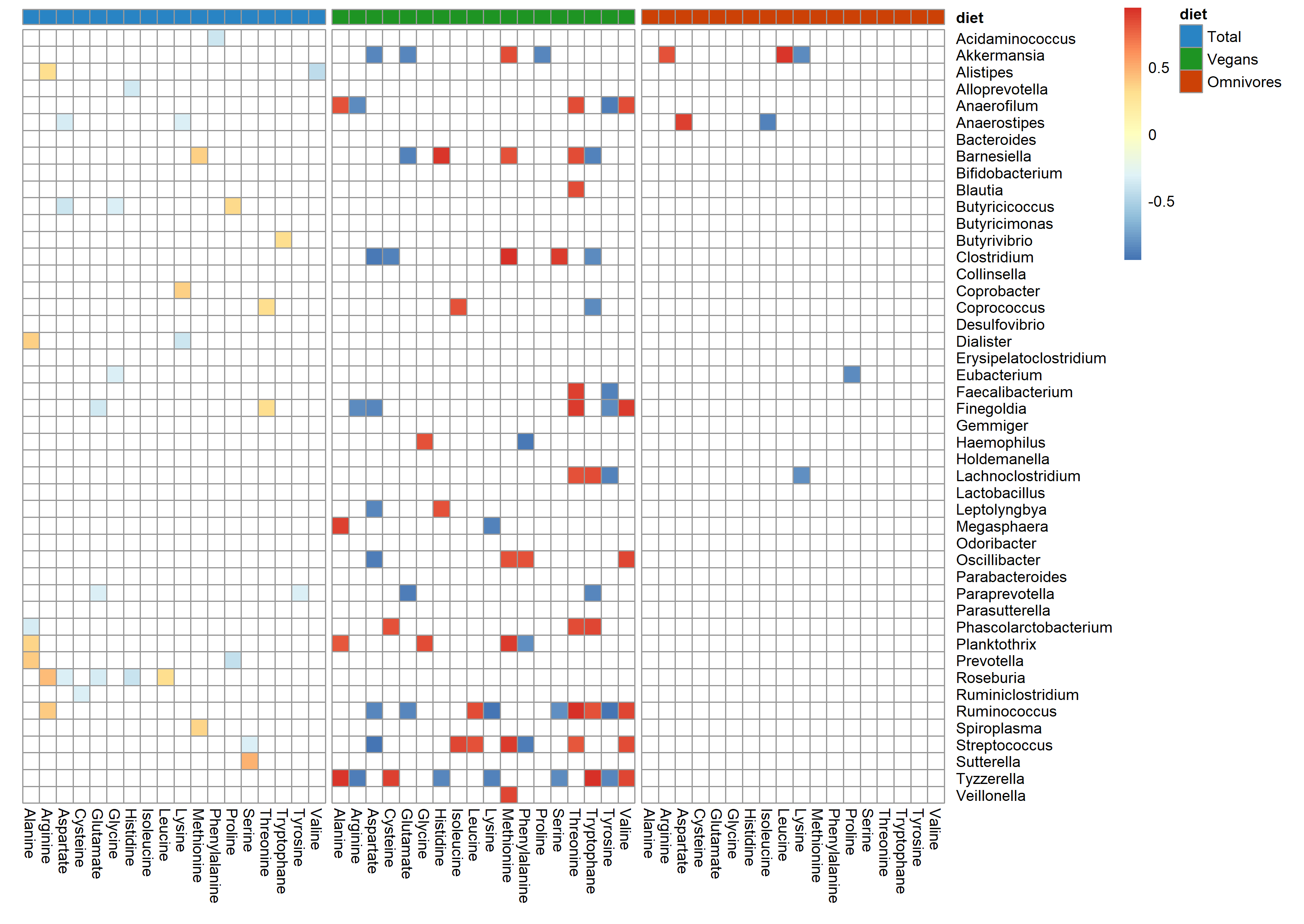


Partial correlations were adjusted for age, sex, body mass index, physical activity, smoking status, alcohol consumption, amino acid supplementation, antibiotics, under-reporting, faecal pH and intake of fibre, carbohydrate and fat. Data of the 36 vegans and 36 omnivores were analysed. The colours in the figure represent partial correlation coefficients from -1 to 1. Shown are only correlation coefficients with uncorrected P-values < 0.05.
